# Lysergic acid diethylamide extends lifespan in* Caenorhabditis elegans*

**DOI:** 10.1007/s10522-026-10521-1

**Published:** 2026-10-22

**Authors:** Beatriz S. Carrilho, Aline Teixeira Duarte Silva, Isabelle Martins, Ariane Moura, Manuela Vilas, Matheus Antonio V. C. Ventura, Francisco Moll, Hugo Aguilaniu, Ivan Domith, Stevens Rehen

**Affiliations:** 1 Institute of Biomedical Sciences, Graduate Program in Morphological Sciences, Federal University of Rio de Janeiro (UFRJ), Rio de Janeiro, 21941-902 Brazil; 2 D’Or Institute for Research and Education (IDOR), Rio de Janeiro, 22281-100 Brazil; 3 Serrapilheira Institute, Rio de Janeiro, Rio de Janeiro 22431-050 Brazil; 4 IDOR Pioneer Science Initiative, Rio de Janeiro, 22281-100 Brazil; 5 Department of Genetics, Institute of Biology, Federal University of Rio de Janeiro (UFRJ), Rio de Janeiro, Brazil

**Keywords:** Psychedelics, Lifespan, Healthspan, *C. elegans*, Nutrient sensing

## Abstract

Aging is modulated by conserved nutrient-sensing pathways that integrate metabolic cues to regulate growth, stress resilience, and lifespan. Caloric restriction (CR) extends longevity across species, motivating the search for compounds that engage related physiology. Lysergic acid diethylamide (LSD), a classic serotonergic psychedelic with emerging therapeutic applications, remains unexplored in the context of aging. Here we show that chronic exposure to nanomolar LSD extends lifespan in *Caenorhabditis elegans* by up to 17.6% at the 25% survival timepoint, and reduces age-associated lipofuscin accumulation. This response involves serotonergic GPCR signaling: LSD failed to extend lifespan in *ser-1* and *ser-5* mutants and shortened lifespan in *ser-4* and *ser-7* mutants, yet remained effective, although with reduced magnitude, in *tph-1* animals deficient in serotonin synthesis, indicating that endogenous serotonin production is not strictly required. LSD also elicited phenotypes that overlap with CR, including reduced reproductive output, smaller body size, delayed vulval development, and time-dependent remodeling of lipid stores and PHA-4 levels, while adult food-consumption assays detected no reduction in feeding under the conditions tested. Notably, the lifespan benefit was absent in *eat-2* mutants and was lost under bacterial dilution, where LSD instead shortened lifespan, indicating that nutritional context shapes the response. Transient exposure during early adulthood was sufficient to extend lifespan. Together, these findings identify LSD as a tool to investigate how serotonergic signaling interfaces with conserved longevity-related pathways.

**Supplementary Information:**

The online version contains supplementary material available at https://doi.org/10.1007/s10522-026-10521-1.

## Introduction

Aging is a complex biological process characterized by the progressive accumulation of sequential biological alterations over time, which increases susceptibility to disease and mortality (Harman 1993). As global population ages, diseases such as cardiovascular conditions, diabetes, Alzheimer’s, and Parkinson’s disease have become increasingly prevalent, posing significant health and socioeconomic challenges. Addressing these challenges requires innovative strategies to extend longevity, delay the aging process, and mitigate the risk of age-associated diseases.

Over the past decades, research has focused on identifying and optimizing interventions to slow aging and promote healthier and longer lives (Hine et al. 2015; Jones et al. 2023). Among these, dietary modifications and regular physical activity have emerged as critical approaches. Specifically, caloric restriction (CR), defined as a reduction of 30–60% of caloric intake without malnutrition, stands out as one of the most effective strategies for extending lifespan and enhancing healthspan (Mercken et al. 2012). CR has been shown to significantly lower the incidence of aging-related diseases such as diabetes, cancer, heart diseases, and neurodegenerative disorders (Colman et al. 2009). Furthermore, fasting, which involves periodic abstinence from food, shares many benefits with CR, enhancing cellular resilience and reducing the risks associated with aging and disease (Longo & Mattson 2014).

The mechanisms underlying the benefits of CR and fasting involve the activation of key molecular pathways, including the insulin/IGF-I-like (IIS) and the target of rapamycin (TOR) pathway. These signaling pathways play crucial roles in cellular protection against oxidative, metabolic, and proteotoxic stress, thereby enhancing longevity and delaying age-associated pathologies (Mattson 2012). However, while the potential of CR to promote healthy aging is well-documented, sustaining a strict regimen in humans is challenging due to social, psychological, and practical constraints. This has led to growing interest in identifying alternative approaches, such as compounds that mimic the effects of CR without requiring drastic dietary restrictions.

Recent research has helped understand the interplay between dietary interventions, serotonin signaling, and aging mechanisms. Serotonin, traditionally recognized for its role in mood regulation, has emerged as a key regulator of aging and longevity (Fidalgo et al. 2013). Studies have shown that serotonin influences lifespan in several organisms, including *Caenorhabditis elegans* (Murakami & Murakami 2007). In murine model, serotonin signaling is intricately linked to pathways such as mTORC1, which mediates the cognitive and metabolic benefits associated with dietary restriction (Teng et al. 2019). Targeting serotonin signaling may represent a novel strategy to promote longevity.

In mammals, serotonergic signaling can be modulated by psychedelics, which have gained significant attention for their potential to promote neuroplasticity, enhance mental health, and influence biological pathways linked to longevity (de Vos et al. 2021). Compounds such as LSD (Lysergic Acid Diethylamide), psilocybin, and N,N-dimethyltryptamine (N,N-DMT), are known to interact with serotonin receptors, impacting neuroplasticity and emotional regulation (Nichols 2016).

Our recent findings highlight the use of *C. elegans* as a model organism for investigating the effects of psychedelics on serotonergic signaling and behavior. In this previous study, we demonstrated that acute LSD exposure reduces locomotion speed in *C. elegans* by 52%, mediated by serotonin receptors SER-1 and SER-4, which are homologous to human serotonin receptors 5HT2 and 5HT1, respectively (Ornelas et al. 2024). Additionally, metabolic profiling revealed that LSD is rapidly absorbed and metabolized into compounds such as Nor-LSD and O-H-LSD, analogous to metabolic pathways in humans (Silveira et al. 2023). While psychedelics have shown protective effects across various physiological systems, their role in modulating aging and promoting longevity remains an underexplored area of research (Inserra et al. 2023; Saeger & Olson 2022).

Given these findings, *C. elegans* emerges as an ideal model for further exploration of the link between serotonin signaling, psychedelics, and aging mechanisms. This study aims to investigate the effects of LSD on longevity. We hypothesize that LSD promotes longevity in *C. elegans* by modulating conserved nutrient-sensing pathways, potentially through mechanisms that overlap with aspects of caloric restriction physiology, thereby delaying physiological markers of aging.

## Methods

### Chemicals

Potassium phosphate monobasic (CAS 7778-77-0, Sigma Aldrich), Sodium phosphate dibasic (CAS 7558-79-4, Sigma Aldrich), Calcium chloride (CAS 10043-52-4, Merck), Magnesium sulfate (CAS 7487-88-9, Sigma Aldrich), Cholesterol (CAS 57–88-5, Sigma Aldrich), Streptomycin sulfate salt (CAS 3810-74-0, Sigma Aldrich), Agar bacteriological (Kasvi), Bacto peptone (Thermo Scientific), Potassium phosphate dibasic (CAS 7758-11-4, Thermo Scientific), LB Broth (Merck), Sodium chloride (CAS 7647-14-5, Merck), LSD (CAS 50-37-3, Lipomed), Oil Red O (CAS 1320-06-5, Sigma Aldrich), PBS (10X), pH 7.4 (Gibco), Triton^™^ X-100 (CAS 9036-19-5, Sigma Aldrich), 5-Fluorouracil (CAS 51-21-8, Sigma Aldrich).

### Maintenance and cultivation of animals

The strains used in this study were obtained from the Caenorhabditis Genetic Center (CGC), University of Minnesota, USA. The strains N2 (Wild type), OH15876 [*pha-4*::3xGAS::GFP::TEV::LoxP::3xFLAG], AQ866 [(*ser-4*(ok512)], DA1814 [*ser-1*(ok345)], VV212 [*ser-5*(vq1)], DA2100 [*ser-7*(tm1325)], MT15434 [*tph-1*(mg280)], DA1116 [*eat-2*(ad1116)], MAH240 [*hlh-30p*::hlh-30::GFP], and VC222 [*raga-1*(ok386)] were cultivated using standard techniques (Brenner 1974). Briefly, the animals were maintained on solid medium in plates prepared with nematode growth medium (NGM) seeded with *Escherichia coli* OP50-1 as a food source and supplemented with 100 μg/L of streptomycin. The plates were kept at 20 °C. To ensure adequate food supply, the animals were regularly transferred to fresh plates.

### Synchronization

The animals were synchronized using the bleaching method according to Porta-de-la Riva *et al.* (Porta-de-la-Riva et al. 2012). In summary, plates containing young adult hermaphrodites and eggs were collected and washed three times with M9 buffer to remove bacterial residues. Then, the bleaching solution (1.65% [v/v] NaOCl, 0.5 M NaOH) was added, and the mixture was agitated to ensure complete lysis of the animals while preserving the eggs. M9 was added to stop the reaction, and the eggs were washed, transferred to bacteria-free plates, and stored at 20 °C for 24 hours. The next day, L1 larvae were transferred to fresh plates containing bacteria to allow the animals to develop.

### Preparation for experiments

Except in the oviposition experiment, 5-fluorouracil was added to the medium at a concentration of 80 µM in long-term assays to prevent egg hatching. This compound interferes with DNA synthesis and blocks cell division. Additionally, to prevent the degradation of the compounds by bacteria, *E. coli* was previously inactivated by heat treatment at 65 °C for 30 minutes.

### Longevity assay

Synchronized animals of the N2, *ser-1*(ok345), *ser-4*(ok512), *ser-5*(vq1), *ser-7*(tm1325), *tph-1*(mg280), *raga-1*(ok386), and *eat-2*(ad1116), strains at the young adult stage (day 0 of adulthood) were transferred to Petri plates containing either 250 nM LSD or vehicle control (water). For the dose-response experiments in N2 animals, worms were continuously exposed to 100, 200, or 250 nM LSD throughout their lifespan. For the mutant strains, in the continuous exposure assay, animals remained on treatment plates throughout their lifespan. Animals were considered dead if they showed no body or head movement upon mechanical stimulation. In parallel, a second longevity assay was performed to evaluate whether the longevity-promoting effects of LSD required continuous exposure. For this experiment, approximately 50 animals were allocated to each condition (Control or LSD 250 nM) and exposed to treatment for 24, 48, 72, 96, or 120 hours, after which both groups were transferred to treatment-free plates. Survival was then monitored every two days using the same scoring criteria.

To investigate whether the lifespan-extending effect of LSD overlaps with a dietary restriction response, an additional longevity assay was performed under reduced bacterial food availability. N2 animals were maintained under either control or 250 nM LSD conditions and provided with a diluted bacterial suspension. The standard bacterial suspension used under *ad libitum* feeding conditions was adjusted to an OD600 of 3.0, whereas the dietary restriction condition consisted of a 10-fold dilution to an OD600 of 0.3. Animals were maintained under these respective feeding and treatment conditions throughout the lifespan assay, and survival was assessed every two days according to the criteria described above.

Survival analysis was performed using OASIS 2, and the results presented correspond to a late-survival time point, defined as the stage at which 25% of the animals remained alive.

### Fluorescence analysis and quantification

Fluorescence from the *pha-4::GFP* and *hlh-30::GFP* reporters were analyzed using the OH15876 and MAH240 strains, respectively, while lipofuscin autofluorescence was assessed in the N2 (wild-type) strain. Synchronized animals at the young adult stage (day 0 of adulthood) were placed on Petri plates containing either 250 nM LSD or control. Each plate corresponded to a specific time point: days 1, 5, 10 or 15 of adulthood. At the end of each treatment period, animals were imaged using a Leica M165 FC stereomicroscope at 120x magnification for *pha-4::GFP* and *hlh-30::GFP* fluorescence, and lipofuscin autofluorescence. Image analysis was performed using LAS X software. For *pha-4::GFP* and lipofuscin, total fluorescence was quantified in the head and in the whole body, respectively, and the fluorescence intensity values were normalized to the corresponding region of interest (ROI) area. In MAH240 animals, HLH-30::GFP nuclear localization was assessed in intestinal cells based on the visual identification of GFP-enriched intestinal nuclei, and animals were classified as positive or negative for nuclear localization. Results were expressed as a percentage relative to the control group.

### Oviposition assessment

Synchronized adult animals were placed on Petri plates containing treatment (LSD 250 nM) or control. At the end of the L4 larval stage, animals were transferred to plates, with one animal per plate. Every 24 hours, the eggs were counted using a Leica Ivesta 3, and the adult animals were transferred to new plates containing the treatment or not, until day 11 of treatment.

### Animal body length

Synchronized animals at the young adult stage (day 0 of adulthood) were placed on plates containing either LSD (250 nM) or control. Animals were assigned to plates corresponding to a single treatment time point (1 or 10 days). At the end of each treatment period, the animals were mounted on 2% agarose pads with 10 mM NaN_3_ on microscope slides. Animal length was measured using LAS X software. Images were acquired using a Leica M165 FC stereomicroscope at 100× magnification.

### Pharyngeal pumping

Synchronized animals at the young adult stage (day 0 of adulthood) were placed on plates containing treatment (LSD 250 nM) or control. Approximately 12 animals were transferred to a plate containing 12 wells (one animal per well). Each well was recorded when the animal reached 1 and 5 days of treatment for 10 seconds using a Leica Ivesta 3 stereomicroscope (Leica, Wetzlar, Germany) with an attached camera and a 110x magnification. The analysis of each video was performed by playing them in slow motion (0.25×) and counting the pharyngeal pumping rate. The number of contractions counted during the 10-second recording period was converted to contractions per minute. Counts were independently assessed by two independent researchers to reduce individual observer bias.

### Food consumption assay

Synchronized animals at the young adult stage (day 0 of adulthood) were placed on NGM plates containing LSD (250 nM) or control. Approximately 20 animals were transferred to each plate and maintained under their respective experimental conditions for 1 and 5 days. Prior to animal transfer, plates were seeded with 600 µL of bacterial suspension concentrated in the center of the NGM surface to minimize bacterial loss to the edges of the plate. At each timepoint, animals were manually removed using a worm pick, and the plates were washed with 3 mL of M9 buffer to recover the bacterial biomass remaining on the NGM surface. The entire recovered suspension was transferred to a conical tube and thoroughly homogenized. Subsequently, 200 µL of each sample was transferred to a 96-well plate in technical triplicate. The remaining bacterial biomass was estimated by measuring the optical density at 600 nm (OD_600_) using a GloMax microplate reader (Promega, Madison, WI, USA).

### Lipid quantification

Synchronized animals at the young adult stage (day 0 of adulthood) were placed on Petri plates containing treatment (LSD 250 nM) or control. Animals were transferred to plates assigned to a single treatment time point (1, 5, or 10 days). Once the treatment ended, lipid quantification was performed according to Nicole Stuhr (Stuhr et al. 2022). In summary, the animals were washed, permeabilized with PBST, and fixed with 60% isopropanol. They were then incubated with Oil Red O 60% for staining. The animals were imaged using a Leica Ivesta 3 stereomicroscope 60x magnification (Leica, Wetzlar, Germany) with the same parameters for all experimental groups.

### Larval development

Synchronized animals at the L1 stage were placed on Petri plates containing treatment (LSD 250 nM) or control. Animals were transferred to each plate, and after 48 hours, the animals were scored for vulval development. The animals were imaged using a Leica SP8 confocal microscope.

### Statistical analysis

Lifespan assays were analyzed by comparing treated and untreated animals of the same strain using the log-rank (Mantel–Cox) test (See supplementary table 1). For all other experiments, statistical significance was assessed using the Student’s *t*-test or Two-way ANOVA, as indicated in the corresponding figure legends and supplementary tables 2, 3, 4, 5, 6, 7 and 8. In all experiments, each n was considered an independent experiment and were performed at least three times. All analyses were performed using GraphPad Prism version 8.0.

## Results

### LSD extends lifespan and reduces lipofuscin accumulation in aging *C. elegans*

**Fig. 1:**
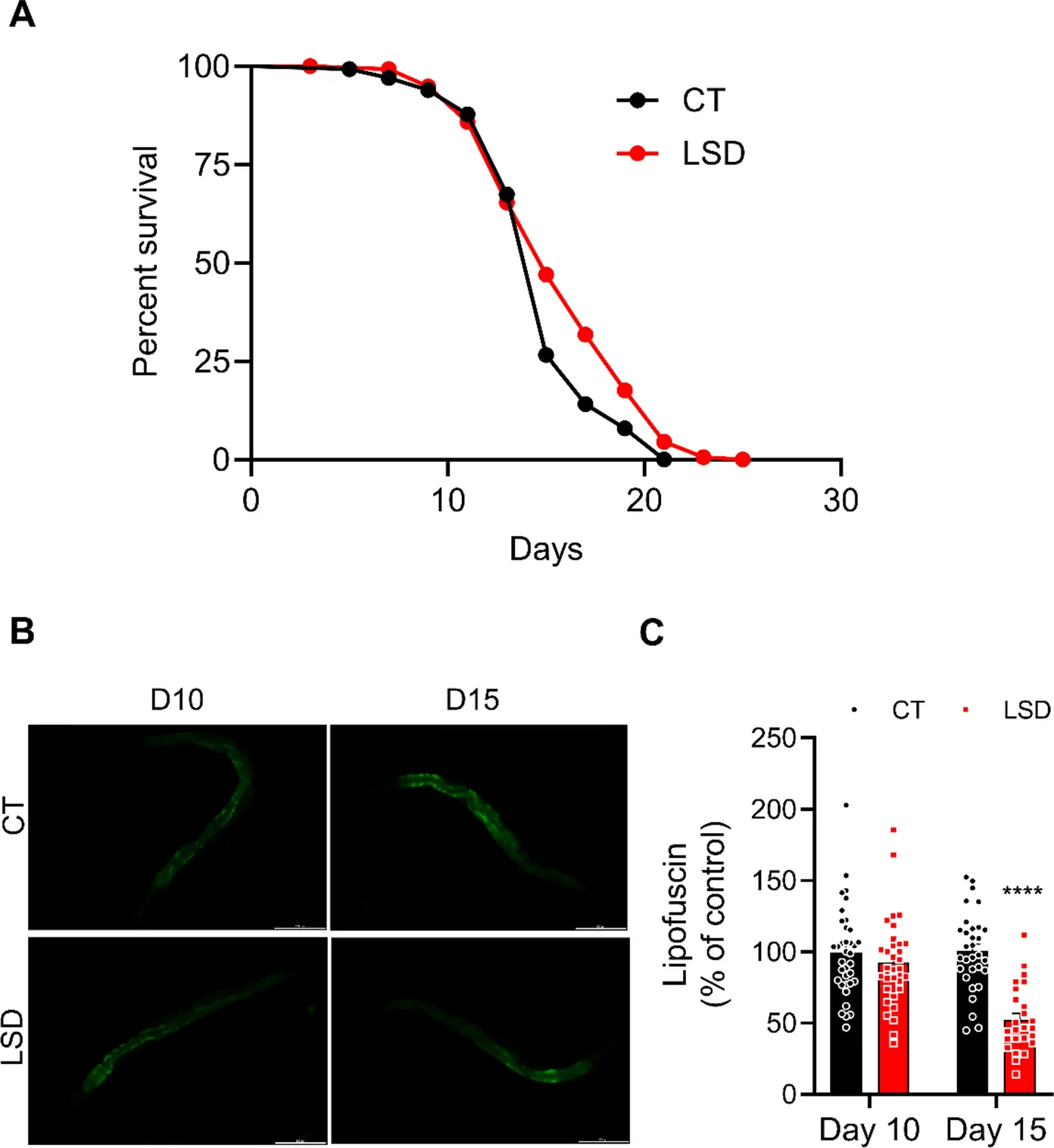
LSD extends the lifespan of *C. elegans* and delays aging. **A** Percentage survival of treated with 250 nM LSD (red) or untreated animals (black) over time. Data presented as Kaplan-Meier survival curves, and group differences analyzed using the Log-rank test. n = 4, with at least 130 animals per condition. **B** Representative images of lipofuscin (green) animals treated (bottom) or untreated (top). Scale bar: 200 µm. **C** Quantification of lipofuscin accumulation in treated with 250 nM LSD (red) or untreated (black) animals on day 10 or 15. Fluorescence intensity expressed as percentage of the control and is presented as mean ± standard error of the mean (SEM). n = 3, with at least 25 animals per condition. Statistical analysis performed using Student’s t-test. ****p<0.0001 compared to the control

Given that serotonergic signaling plays a crucial role in modulating longevity in *C. elegans* (Murakami & Murakami 2007), we investigated whether chronic exposure to LSD increases nematode longevity. First, we performed a lifespan dose–response analysis to define the concentration used in subsequent experiments. Treatment with LSD at 100, 200, and 250 nM increased lifespan by 12.4, 13.2, and 17.6%, respectively, compared with controls (Supplementary table 1 and fig. 1). Since multiple doses were active in the nanomolar range, 250 nM was selected as the working concentration for subsequent experiments (Fig. 1a).

Although studying longevity provides insights into aging, increased lifespan does not necessarily reflect an extension of healthspan. To determine whether LSD also delays aging, we quantified lipofuscin, a fluorescent pigment that accumulates due to the incomplete degradation of cellular components. As its accumulation progressively increases with age, lipofuscin is considered a reliable marker of aging (Porta 2002). We analyzed 10- and 15-day-old worms, as these time points allow sufficient lipofuscin accumulation to detect aging-related changes. LSD significantly reduced lipofuscin accumulation on day 15 compared with control, suggesting a delay in age-associated decline in addition to its lifespan-extending effect. (Day 10: CT 100% ± 10.5, LSD 92.5% ± 5.6; Day 15: CT 100% ± 6.4, LSD 49.8% ± 6.3, p < 0.0001) (Fig. 1b, c and supplementary table 6).

### LSD-induced longevity depends on serotonergic GPCR signaling but not endogenous serotonin synthesis

**Fig. 2:**
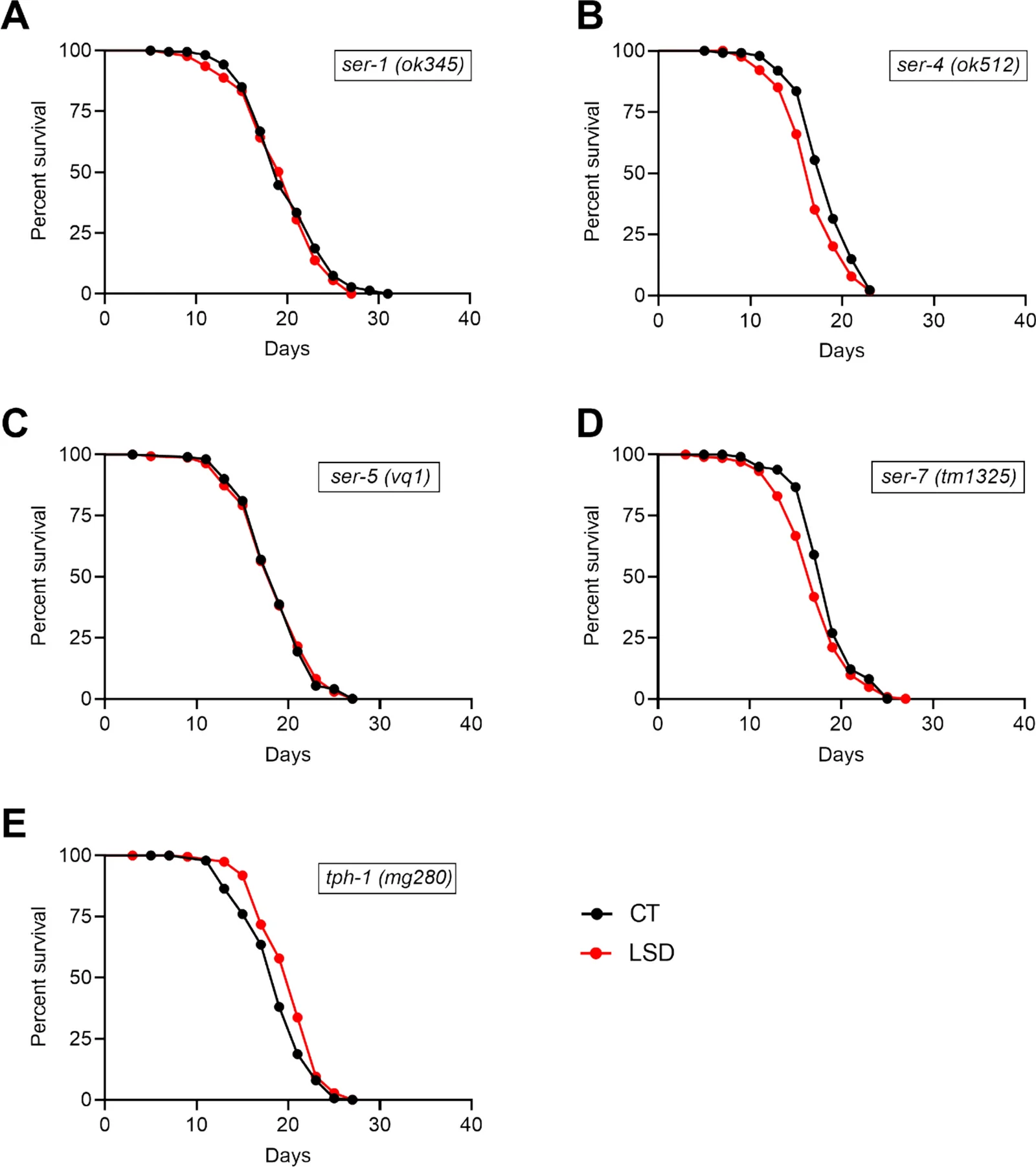
LSD-induced longevity depends on serotonergic GPCR signaling but not endogenous serotonin synthesis. Percentage of survival of mutant animals *ser-1* KO (**A**), *ser-4* KO (**B**), *ser-5* KO (**C**), *ser-7* KO (**D**) and *tph-1* KO (**E**) treated with 250 nM LSD (red) or untreated (black) over time. All data presented as Kaplan-Meier survival curves, and group differences analyzed using the Log-rank test. *ser-1* and *ser-4*: n = 4; *ser-5*, *ser-7* and *tph-1*: n = 3 At least 120 animals per condition

In our previous work, we identified SER-1 and SER-4 as key receptors involved in the behavioral changes triggered by LSD (Ornelas et al. 2024). Building on these findings, we investigated whether serotonergic receptors also contribute to LSD-induced longevity. In *ser-1* and *ser-5* mutants, LSD failed to significantly extend lifespan compared with their respective controls. In contrast, LSD significantly shortened lifespan in both *ser-4* and *ser-7* mutants, by 7.7 and 3.3%, respectively (Fig. 2a–d and Supplementary Table 1). Together, these findings show that disruption of multiple serotonergic receptors alters the lifespan response to LSD. The absence of lifespan extension in *ser-1* and *ser-5* mutants, together with the significant lifespan reduction observed in *ser-4* and *ser-7* mutants, supports an involvement of serotonergic GPCR signaling in modulating LSD-associated longevity.

To determine whether endogenous serotonin synthesis itself is required for this effect, we next evaluated *tph-1* mutants, which lack tryptophan hydroxylase and are therefore deficient in serotonin synthesis. In contrast to the serotonergic receptor mutants, LSD treatment still significantly extended lifespan in *tph-1* animals by 9.3%, although the magnitude of the effect was reduced compared with that observed in wild-type animals (Fig. 2e and Supplementary Table 1). These findings indicate that endogenous serotonin synthesis is not strictly required for LSD-induced lifespan extension, while suggesting that serotonin availability may modulate the magnitude of the response.

### LSD induces phenotypes that overlap with caloric restriction

In *C. elegans*, serotonergic signaling is not only involved in locomotor responses to food availability, such as the enhanced slowing response (Chase & Koelle 2007), but also plays a broader role in nutrient-sensing and metabolic regulation. Since our previous work showed that LSD can elicit this response (Ornelas et al. 2024), we hypothesized that LSD-treated animals might display additional characteristics associated with CR.

**Fig. 3:**
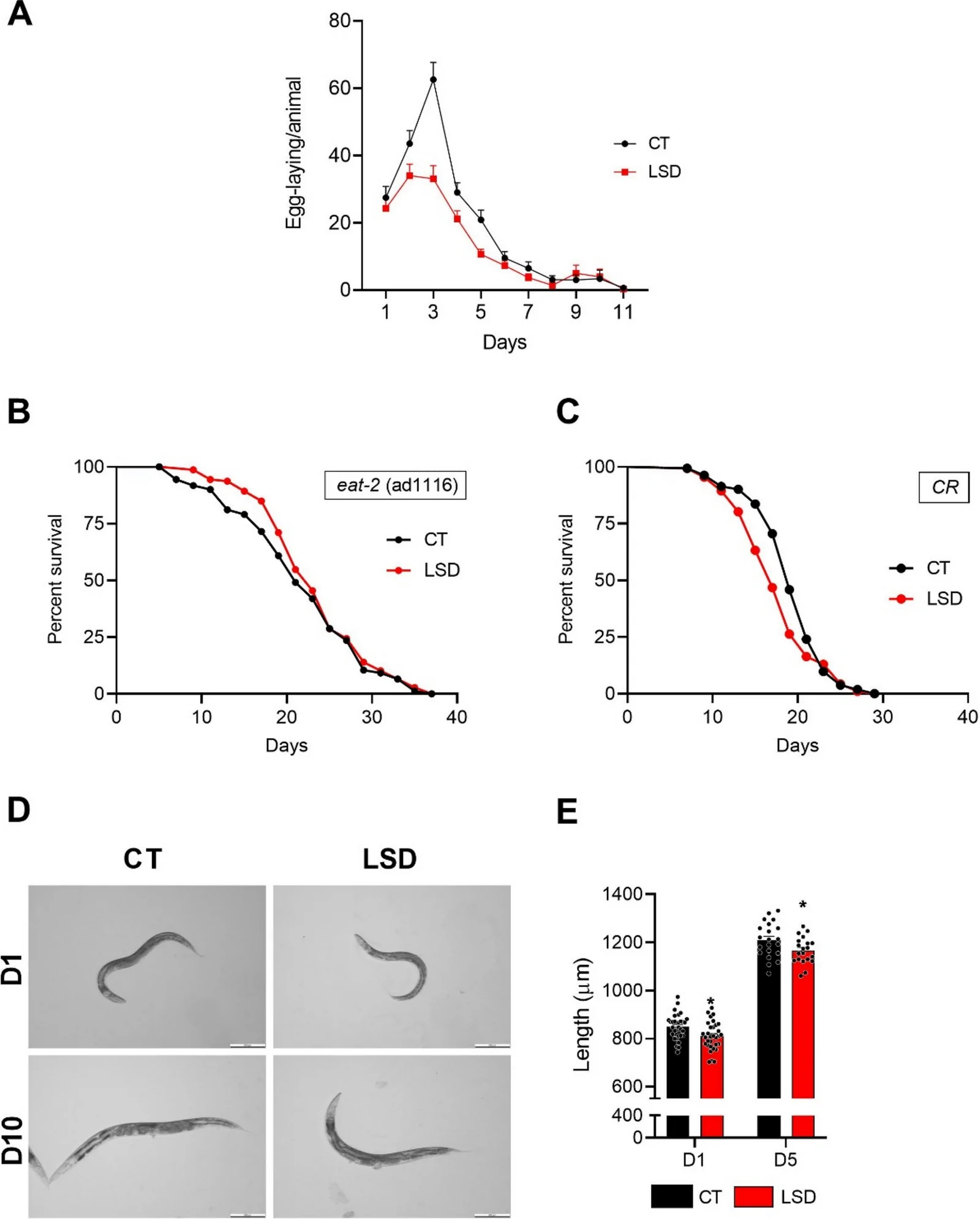
LSD induces phenotypes that overlap with caloric restriction*.* **A** Number of eggs laid during the reproductive period by treated animals with 250 nM LSD (red) or untreated animals (black) per animal/day. Data presented as mean ± standard error of the mean (SEM). n = 5. Statistical analysis performed using Two-way ANOVA interaction, ****p<0.0001 compared to the control. **B** Percentage of survival of mutant animals (DA1116 *eat-2*) treated with LSD 250 nM (red) or untreated (black) over time. All data presented as Kaplan-Meier survival curves, and group differences analyzed using the Log-rank test. n = 4, with at least 130 animals per condition. **C** Percentage of survival of wild type animals subjected to caloric restriction treated with LSD 250 nM (red) or untreated (black) over time. All data presented as Kaplan-Meier survival curves, and group differences analyzed using the Log-rank test. n = 3, with at least 120 animals per condition**. D** Representative images of body length for treated (right) and untreated (left) animals over time. Scale bar: 200 µm. **E** Measurement of body length of treated animals with 250 nM LSD (red) or untreated animals (black) over time. Each point represents an individual animal. Statistical analysis was performed using Student’s t-test. *p<0.05 compared to the control

A well-established consequence of CR in *C. elegans* is reduced oviposition (Hibshman et al. 2016). We quantified egg-laying in LSD-treated animals and observed that the effect of treatment varied over time (two-way ANOVA interaction, p<0.0001; Fig. 3a and supplementary table 2), suggesting a CR-like modulation of reproductive output. CR is also known to reduce body size in *C. elegans* (Hibshman et al. 2016). We measured the length of treated and untreated animals on day 1 and 10 and identified that LSD treatment leads to a significant reduction in body length at all time points (Day 1: CT 849.5 µm ± 9.17, LSD 810.0 µm ± 10.27, p<0.01; Day 10: CT 1210.0 µm ± 15.86, LSD 1166.0 µm ± 12.37, p<0.05) (Fig. 3d, e and supplementary table 3). A classical approach to induce dietary restriction in *C. elegans* is through genetic models with reduced pharyngeal pumping, such as the *eat-2* (ad1116) strain. To assess the effect of LSD treatment in this context, we used this mutant in lifespan assays. No differences in survival were observed between control and treated animals (Fig. 3b and supplementary table 1). To further evaluate the effect of LSD under dietary restriction using a non-genetic approach, we performed lifespan assays under reduced food availability by providing a 10-fold diluted bacterial food source. Under these conditions, LSD treatment did not extend lifespan compared with the dietary-restricted control group. Instead, LSD treatment reduced lifespan by 9.2% (Fig. 3c and supplementary table 1). Together, these results show that the lifespan-extending effect of LSD is lost under both genetic and food-dilution models of dietary restriction.

### LSD modulates caloric restriction-associated and metabolic outcomes over time without decreasing pharyngeal pumping

**Fig. 4:**
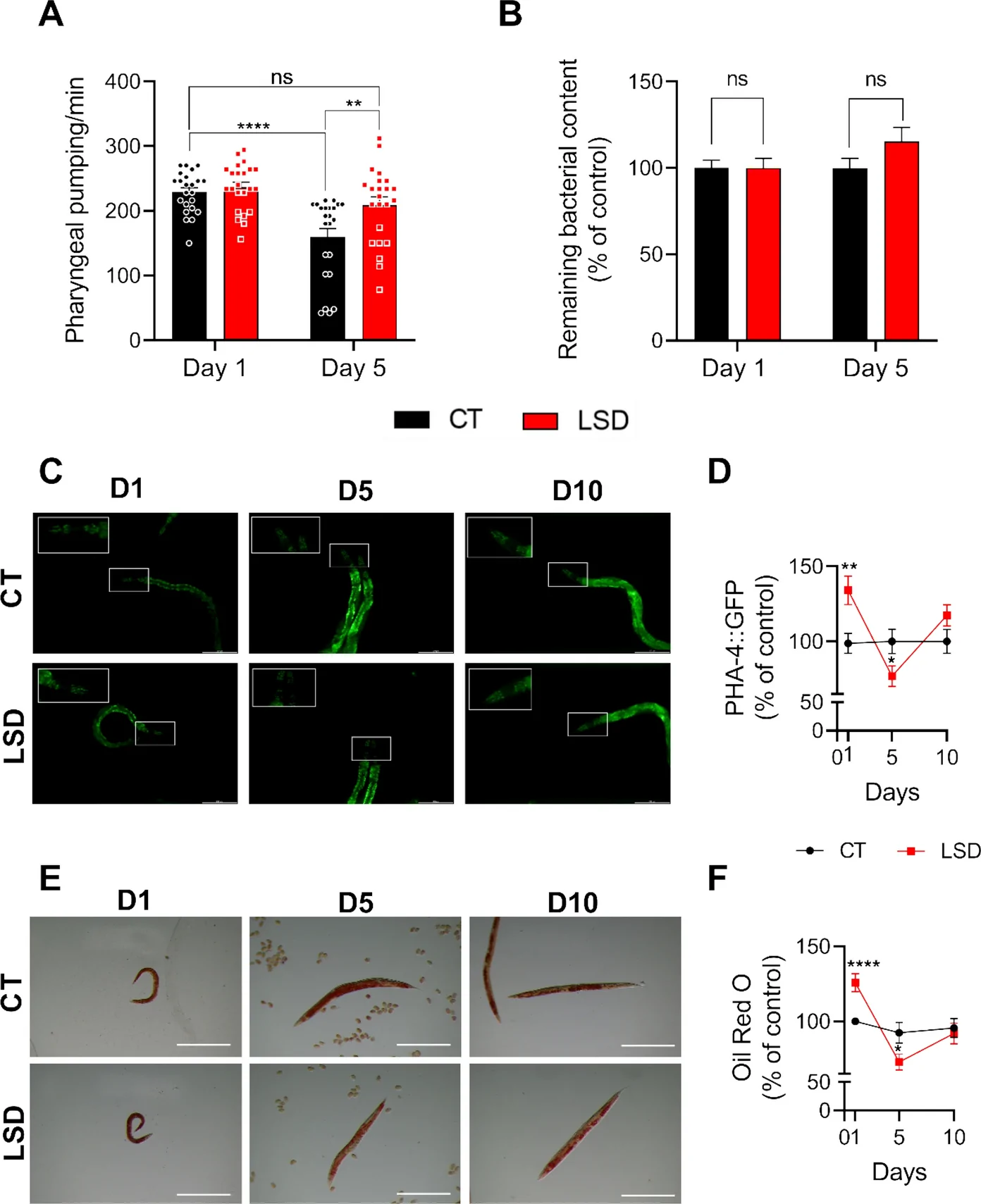
LSD modulates caloric restriction-associated and metabolic outcomes over time without decreasing pharyngeal pumping. **A** Quantification of pharyngeal pumping in treated with LSD 250 nM (red) or untreated animals (black) per minute. Each point represents an individual animal. Data presented as mean ± standard error of the mean (SEM). n = 3. Statistical analysis was performed using two-way ANOVA followed by Sidak’s multiple comparisons test. **p<0.01; ****p<0.0001. **B** Quantification of food consumption in animals treated with LSD 250 nM (red) or untreated animals (black), assessed by remaining bacterial concentration at days 1 and 5 of adulthood. Remaining bacterial content was quantified by measuring optical density at 600 nm (OD_600_). Data are presented as mean ± standard error of the mean (SEM). n = 4 independent experiments. Statistical analysis was performed using Student’s t-test. **C** Representative images of untreated (left) or treated (right) PHA-4::GFP animals, with the insert in the upper left corner indicating the head region selected for fluorescence analysis. Scale bar: 200 µm **D** Quantification of PHA-4::GFP fluorescence intensity in the head region of treated (red) or untreated (black) animals. n = 3 **E** Representative images of treated (right) and untreated (left) animals stained with Oil Red O (ORO) over time. Scale bar: 0.5 mm. **F** Quantification of total lipids in treated (red) or untreated animals (black) through ORO staining represented by % of control D1. n = 3. **D**;**F** All data presented as mean ± standard error of the mean (SEM). Statistical analysis performed using Student’s t-test. *p<0.05; **p<0.01; ****p<0.0001 compared to the control

In wild-type animals, CR results from reduced food intake and is associated with adaptive changes that influence longevity. To assess whether LSD could indirectly trigger a CR-like response by reducing food intake, we measured the pharyngeal pumping rate of treated animals. Analysis on days 1 and 5 of adulthood revealed that LSD increased pharyngeal pumping frequency only on day 5 (Day 1: CT 229.0 ± 6.6, LSD 236.3 ± 7.6; Day 5: CT 159.4 ± 13.2, LSD 209.2 ± 12.2) (Fig. 4a and Supplementary Table 4).

To directly evaluate food consumption, we also performed a food consumption assay. No significant differences in bacterial consumption were observed between control and LSD-treated animals on either day 1 or day 5 (Fig. 4b and supplementary table 5). These findings argue against a simple reduction in food intake as the basis for the CR-like phenotypes observed previously and instead suggest that LSD engages aspects of nutrient-sensing physiology without suppressing feeding behavior under the conditions tested.

Different dietary restriction paradigms can engage distinct cell signaling (Greer & Brunet 2009). We therefore examined downstream readouts commonly associated with CR and nutrient-sensing physiology, including parameters linked to TOR signaling, and assessed whether LSD treatment modulates PHA-4 levels, a transcription factor acting downstream of TOR signaling, as well as lipid regulation. At day 1 of adulthood, treated animals displayed increased PHA-4 levels compared to control. This was followed by a reduction on day 5, while by day 10, levels were comparable to those of untreated animals (Day 1: CT 100.0 ± 6.5, LSD 134.2 ± 9.5, p<0.01; Day 5: CT 100.0 ± 8.11, LSD 76.9 ± 6.93 p<0.05; Day 10: CT 100.0 ± 7.96, LSD 117.4 ± 7.1) (Fig. 4c and 4 d, Supplemental Table 6).

Similarly, lipid accumulation was dynamically regulated by LSD treatment. An increase in lipid content was observed on day 1, followed by a decrease on day 5, and a return to baseline levels by day 10 (Fig. 4e and 4f, Supplemental Table 7; Day 1: CT 100% ± 3.0, LSD 125.7% ± 6.1, p<0.0001; Day 5: CT 90.3% ± 7.9, LSD 70.2% ± 5.8, p<0.05; Day 10: CT 93.1% ± 7.9, LSD 90.4% ± 10.1). Together, these findings reveal a transient and time-dependent metabolic remodeling in LSD-treated animals.

### LSD alters readouts commonly associated with nutrient-sensing pathways in *C. elegans*

**Fig. 5:**
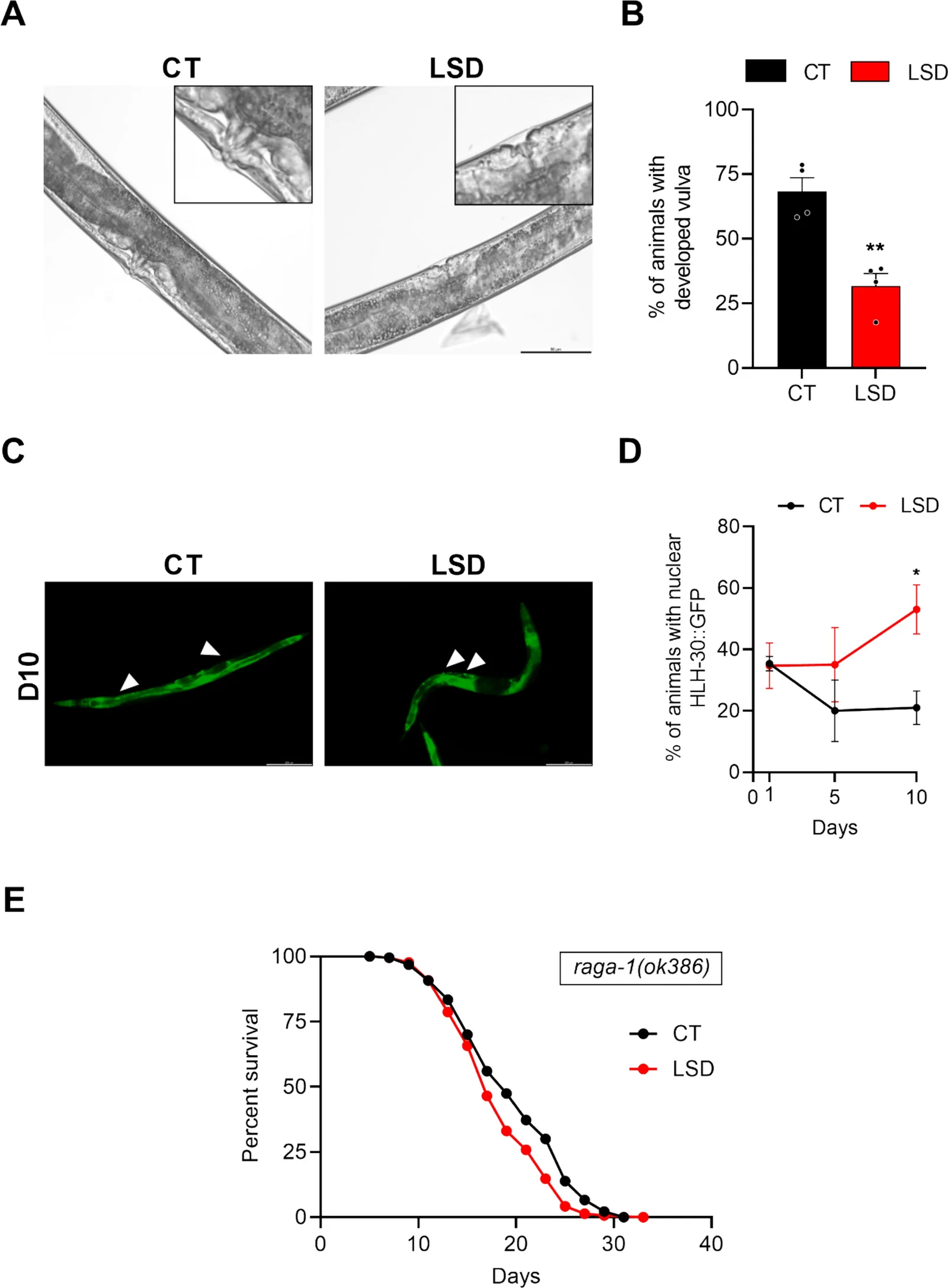
LSD affects developmental and transcriptional parameters associated with TOR signaling*.* **A** Representative image of the vulva (insert) in treated with LSD 250 nM (right) or untreated (left) animals. Scale bar: 50 µm. **B** Percentage of treated (red) or untreated (black) animals with developed vulvas at the end of the L4 larval stage. Each point represents an experimental n. n = 4. Statistical analysis performed using Student’s t-test. **p<0.01 compared to the control **C** Representative images of HLH-30::GFP animals, treated (right) or untreated (left). Scale bar: 200 µm. **D** Quantification of animals displaying nuclear HLH-30::GFP localization in treated (red) or untreated animals (black). n = 3. Statistical analysis performed using Student’s t-test. *p<0.05 compared to the control. **E** Survival percentage of mutant animals (*raga-1* KO) treated with LSD 250 nM (red) or untreated (black) over time. All data presented as Kaplan-Meier survival curves, and group differences analyzed using the Log-rank test. n = 4, with at least 160 animals per condition

To further explore cellular pathways that might contribute to the effects of LSD, we examined several developmental and transcriptional readouts previously linked to TOR pathway. As TOR signaling has been implicated in developmental timing, we assessed vulval formation in young adult nematodes to determine whether LSD induces a delay in larval growth (Jia et al. 2004). For this, we treated animals from the L1 stage and monitored until complete vulval maturation in young adults. Our results indicate that the number of animals with fully developed vulva was significantly lower in the LSD-treated group (CT 68.3% ± 5.3, LSD 31.7% ± 4.8, p<0.01) (Fig. 5a and b, Supplemental Table 8). This result is consistent with altered developmental timing and is compatible with modulation of TOR-related processes.

Next, we examined HLH-30/TFEB nuclear localization, as this transcription factor accumulates in the nucleus under conditions of reduced TOR activity (Silvestrini et al. 2018). LSD treatment increased the number of animals with nuclear translocation on day 10, but not on days 1 and 5 (Day 1: CT 35.3 ± 2.3, LSD 34.7 ± 7.4; Day 5: CT 20.0 ± 10.0, LSD 35.0 ± 12.1; Day 10: CT 21 ± 5.5, LSD 53.0 ± 8.0, p<0.05) (Fig. 5c and d, Supplemental Table 6). Finally, we performed a lifespan assay using the VC222 [*raga-1(ok386)*] strain, a deletion mutant affecting the Rag GTPase required for TORC1 activation. In contrast to the lifespan extension observed in wild-type animals, LSD treatment significantly reduced lifespan by 11.6% in *raga-1* mutants compared with controls (Fig. 5e and Supplementary Table 1). This opposite response suggests that *raga-1* status influences the effect of LSD on lifespan. These findings are consistent with a possible involvement of RAGA-1/TOR-associated processes in the longevity response to LSD, but they do not establish that LSD directly alters TORC1 activity or that TORC1 mediates the effect. Together, these data are consistent with chronic LSD engaging TOR-associated processes, but do not by themselves demonstrate reduced TORC1 activity.

### LSD-induced longevity does not require continuous exposure

**Fig. 6:**
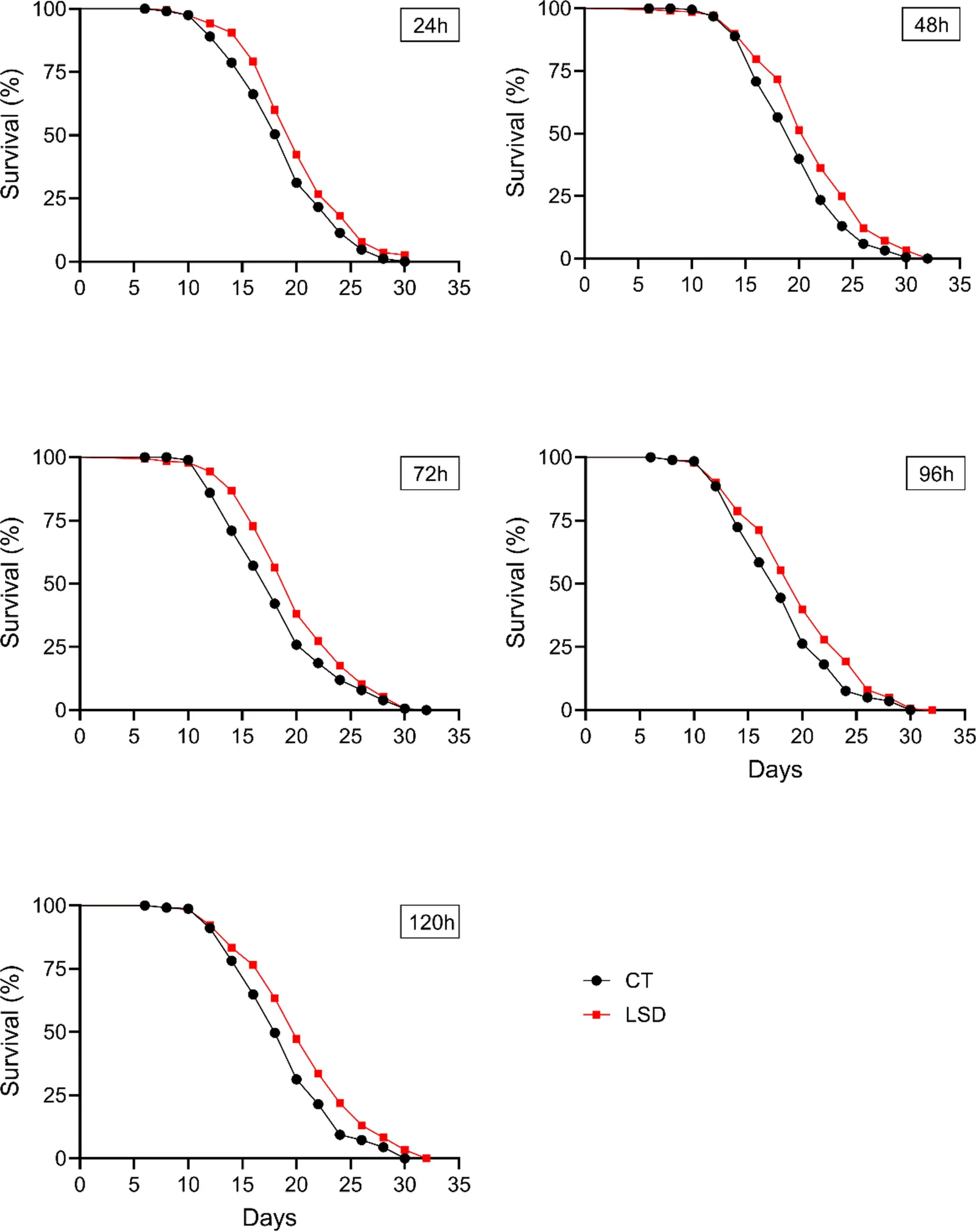
LSD-induced longevity does not require continuous exposure. Percentage of survival of animals treated with LSD 250 nM for 24, 48, 72, 96 or 120 hours (red) or untreated (black) over time. All data presented as Kaplan-Meier survival curves, and group differences analyzed using the Log-rank test. n = 4, with at least 180 animals per condition

Because several LSD-induced physiological and metabolic changes were most evident during the first days of adulthood, we tested whether limiting exposure to this initial period would be sufficient to affect lifespan. Animals were therefore treated with 250 nM LSD for 24, 48, 72, 96, or 120 hours and then maintained in the absence of the compound for the remainder of life (Fig. 6 and Supplemental Table 1). Under these conditions, all exposure time extended longevity (24 h: 5.6%; 48 h: 9.9%; 72 h: 11.0%; 96 h: 12.1%; 120 h: 11.1%). These results suggest that brief early-adulthood exposure can be sufficient to confer a longevity benefit; however, direct clearance controls are required to exclude residual exposure. Notably, the effect was detectable across multiple portions of the survival curve, suggesting that the benefit was not restricted to late survival alone.

## Discussion

In this work, we show that LSD promotes lifespan extension while reducing lipofuscin accumulation, suggesting beneficial effects on both longevity and aging-related decline. These findings are consistent with an emerging view that psychedelics may influence core aging-related processes. Supporting this idea, a recent study reported that psilocybin/psilocin extended cellular lifespan in human fibroblasts and improved survival in aged mice, alongside reductions in senescence and oxidative stress-associated markers (Kato et al. 2025).

In our previous study, we showed that the behavioral slowing effect of LSD in *C. elegans* was absent in animals lacking serotonin receptors SER-1 and SER-4, indicating that both are involved in mediating its neuromodulatory actions (Ornelas et al. 2024). Building on those findings, we asked whether serotonergic receptors also play a role in the longevity effects induced by LSD. In *ser-1* mutants, LSD failed to extend lifespan, supporting the involvement of SER-1 in the pro-longevity effect of LSD. In contrast, *ser-4* mutants showed a lifespan reduction upon LSD treatment, suggesting that, in the absence of SER-4, LSD may exert a deleterious effect.

We further extended this analysis to SER-5 and SER-7 receptors, the other two canonical serotonergic GPCRs in *C. elegans*. Similar to *ser-1*, LSD failed to significantly extend lifespan in *ser-5* mutants, while significant lifespan reduction was observed in *ser-7*, indicating that both receptors also contribute to the pro-longevity response. Taken together, these findings suggest that the lifespan-extending effect of LSD is not mediated by a single serotonin receptor subtype, but may instead depend on the integrity of multiple serotonergic GPCR pathways. Dependence on multiple serotonin receptors is not unprecedented in C. elegans. Serotonergic control of several physiological and behavioral outputs emerges from the coordinated activity of multiple receptor subtypes rather than from a single receptor acting in isolation. For example, comprehensive genetic analysis of serotonin-induced locomotor slowing showed that the response depends on the integrated activity of several serotonin receptors, with SER-4 acting as a major driver and SER-1, SER-5, and SER-7 providing additional modulatory contributions (Dag et al. 2023).

In contrast, a different pattern was observed in *tph-1* mutants, which are deficient in serotonin synthesis. In these animals, LSD was still able to significantly extend lifespan, although the magnitude of the effect was reduced compared with wild-type animals. This finding suggests that endogenous serotonin production is not strictly required for the pro-longevity action of LSD, despite the clear requirement for serotonergic receptors. Thus, LSD may act directly or indirectly through serotonergic GPCR signaling even under conditions of reduced endogenous serotonin availability, while endogenous serotonin may modulate the magnitude of the response.

This distinction is particularly notable because SER-1, SER-4, SER-5, and SER-7 constitute the four canonical serotonergic GPCRs in *C. elegans* and couple to distinct downstream G-protein signaling pathways. Therefore, the loss of the longevity effect across these receptor mutants, together with its persistence in *tph-1* mutants, suggests that LSD-induced lifespan extension depends more strongly on the organization and integration of serotonergic receptor signaling than on serotonin synthesis itself. The distinct phenotype observed in *ser-4* mutants further indicates that the contribution of each receptor is not necessarily equivalent. Altogether, these results support a model in which LSD-induced longevity may depend on coordinated serotonergic GPCR signaling rather than a generic increase in serotonergic tone.

The longevity effects of LSD exhibit parallels with those of serotonergic transporter inhibitors such as fluoxetine, paroxetine, and sertraline, which extend lifespan in *C. elegans* by modulating serotonin receptor pathways (Liang et al. 2024; C. Zhou et al. 2024; Y. Zhou et al. 2024). Notably, the serotonin antagonist mianserin has been proposed to mimic CR, a phenomenon wherein lifespan extension is achieved without necessarily reducing nutrient intake, by engaging serotonin receptor-mediated signaling (Petrascheck et al. 2007). Similarly, LSD may share some of these effects, as our results suggest the modulation of features compatible with CR, including reduced egg laying (Hughes et al. 2007) and body size (Hibshman et al. 2016), as well as the absence of an additive effect in lifespan assays using the *eat-2* strain.

To further examine this relationship using a dietary restriction paradigm independent of the *eat-2* genetic model, we performed lifespan assays under reduced food availability by providing a 10-fold diluted bacterial food source. Under these conditions, LSD not only failed to extend lifespan relative to the dietary-restricted control group but instead resulted in a shorter lifespan.

A comparable pattern has previously been reported for metformin, a compound proposed to induce a dietary restriction-like state in *C. elegans*. While metformin extends lifespan in well-fed wild-type animals, it fails to further extend lifespan in *eat-2* mutants and, at effective pro-longevity doses, can even shorten lifespan in this dietary restriction background (Onken & Driscoll 2010).

The authors proposed that further stimulation of CR-associated physiology in animals already experiencing dietary restriction may produce diminishing or even detrimental effects. This observation closely parallels the loss of LSD-mediated lifespan extension under both *eat-2* and reduced-food conditions. Taken together, these findings are consistent with a partial overlap between the effects of LSD and physiological programs associated with CR.

Importantly, however, this pattern does not appear to result from a direct reduction in feeding capacity. On the first day of adulthood, LSD treatment did not significantly alter pharyngeal pumping activity. By day 5, although treated animals exhibited a higher pumping rate than control, their pumping activity did not differ from that observed in control animals on day 1 of adulthood. Considering that pharyngeal function progressively declines during aging in *C. elegans* (Cho et al. 2025), these findings suggest that LSD does not increase pumping above the physiological levels observed in young animals, but may instead contribute to the preservation of this function during aging. This interpretation is also consistent with the reduced accumulation of lipofuscin observed in treated animals at later ages, suggesting a possible attenuation of age-associated physiological decline.

Furthermore, food consumption, assessed by quantifying the residual bacterial biomass after exposure to the animals, did not differ significantly between control and LSD-treated groups on either day 1 or day 5. Thus, the higher pharyngeal pumping observed in LSD-treated animals at day 5 was not accompanied by a measurable increase in overall food consumption. This dissociation further supports the interpretation that LSD may preserve pharyngeal function during aging without substantially altering feeding behavior under the conditions tested.

The preservation of pharyngeal pumping during aging does not appear to be unique to LSD and has also been reported for other compounds. The effects of allantoin, trichostatin A (TSA), rapamycin, and LY-294002 on pharyngeal function were evaluated throughout adulthood in *C. elegans*, with measurements performed on days 1, 5, 10, and 15 (Calvert et al. 2016). Although pharyngeal pumping progressively declined with age in all groups, this decline was attenuated in treated animals. These findings were interpreted as evidence of preserved physiological function during aging rather than an increase in food intake per se. Similarly, our results raise the possibility that the higher pharyngeal pumping observed in LSD-treated animals on day 5 reflects maintenance of feeding capacity at an age when this function has already begun to decline in control animals, further supporting a potential beneficial effect of LSD on healthspan.

In many dietary restriction paradigms in *C. elegans*, increased longevity is accompanied by lipid metabolic remodeling and, frequently, by a reduction in fat content during adulthood. However, this response is not uniform and may vary depending on the protocol, the developmental stage analyzed, and the severity of the restriction (Palgunow et al. 2012). Indeed, different CR interventions engage distinct metabolic pathways, and in some contexts, dietary restriction can even lead to a transient increase in triglyceride levels and expansion of lipid droplets (Greer & Brunet 2009). In this context, the increase in lipid content observed after 1 day of LSD treatment may reflect an early phase of metabolic reorganization. The reversal of this trend by day 5, when lipid levels decrease, suggests that the treatment induces a dynamic and time-dependent response consistent with a progressive adaptation of energy metabolism.

In parallel, PHA-4 expression appeared to follow a similar pattern, with an increase on the first day of treatment followed by a reduction on the fifth day. Previous studies have demonstrated the essential role of this transcription factor in lifespan extension associated with dietary restriction, involving both its nuclear activity and increased expression (Panowski et al. 2007; Sheaffer et al. 2008). In addition, PHA-4 has been shown to promote lipid accumulation under conditions of nucleolar stress through the transcriptional activation of lipogenic genes (Wu et al. 2018). In this context, the increase in PHA-4 observed after 1 day of LSD treatment is compatible with an early response that overlaps with nutrient-sensing (Panowski et al. 2007) or anabolic-state remodeling, although these data do not distinguish between these possibilities (Sheaffer et al. 2008), even in the absence of a detectable reduction in food intake. At the same time, this early increase in PHA-4 may also help explain the transient rise in lipid content observed at day 1, consistent with a phase of metabolic reorganization (Wu et al. 2018). In turn, the decrease observed on day 5 may indicate that this initial response is not sustained at the same level over time, which is consistent with a transient physiological adaptation (Cornwell et al. 2024), followed by the establishment of a new state of equilibrium.

Although LSD exhibits features consistent with a CR-like response, its effects may not fully overlap with those of classical CR mimetic agents. For example, rapamycin, which also modulates nutrient-sensing pathways via mTOR inhibition, is associated with adverse effects such as glucose intolerance and increased diabetes risk (Unnikrishnan et al. 2020). Psychedelics, in contrast, have been associated with reduced metabolic risks, such as protective effects on pancreatic β-cells, lower odds of diabetes and heart diseases (Gojani et al. 2024; Simonsson et al. 2021). However, these observations should not be taken as direct evidence that LSD reproduces the full physiological profile of CR mimetics. In the context of the present study, the main conclusion is more limited: LSD induces a physiological program in *C. elegans* that shares several features with CR-associated responses.

An alternative interpretation of our findings is that LSD may act, at least in part, as a mild stressor, thereby eliciting a hormetic response. Mild cellular stress can activate adaptive pathways involved in stress resistance, proteostasis, autophagy, and metabolic remodeling, ultimately contributing to improved survival and longevity in many species, including *C. elegans* (Kumsta et al. 2017). The absence or reversal of lifespan extension in several serotonergic mutants, in *raga-1* mutants, and under reduced bacterial availability suggests that the effect of LSD is strongly context dependent and may become neutral or detrimental under sensitized genetic or nutritional conditions. At the same time, the persistence of lifespan extension in *tph-1* mutants suggests that endogenous serotonin synthesis is not strictly required, whereas the altered responses observed in multiple serotonergic GPCR mutants are consistent with a contribution of serotonergic receptor signaling. These findings therefore support the possibility of a receptor-dependent adaptive stress response, although they do not distinguish this model from a more direct contribution of serotonergic and nutrient-sensing pathways to longevity.

Furthermore, TOR pathway plays a pivotal role in growth, metabolism, and aging (Keith Blackwell et al. 2019). In our study, LSD delayed larval development, reduced body size and increase of HLH-30 nuclear translocation at later time point. Together, these observations are compatible with modulation of TOR-associated processes, as described in dietary restriction and rapamycin paradigms (Bar et al. 2016; Duong et al. 2020; Keith Blackwell et al. 2019). Consistent with this interpretation, the lifespan response to LSD was altered in *raga-1*(ok386) mutants. Whereas LSD extended lifespan in wild-type animals, treatment significantly reduced lifespan by 11.6% in *raga-1* mutants, suggesting that the genetic status of the RAGA-1/TOR axis influences the longevity response to LSD. However, psychedelics like LSD and DMT activate 5-HT2A receptors, which have been shown to mediate mTOR activation during short-term treatments, such as those enhancing neuroplasticity in cortical neurons (Ly et al. 2018; N. Costa et al. 2024). Proteomic analyses of overnight LSD-treated human cerebral organoids reveal upregulation of proteins involved in mTOR signaling and synaptic plasticity, further supporting this mechanism during acute exposure (N. Costa et al. 2024). Whether prolonged LSD exposure produces signaling adaptations distinct from acute exposure remains to be determined. Studies on 5-HT6 receptor signaling demonstrate that receptor desensitization and decreased membrane expression attenuate mTORC1 activity over time, a phenomenon that may also apply to 5-HT2A receptors with prolonged LSD exposure (Teng et al. 2019). Our data are consistent with a temporal shift in TOR-related readouts, but they do not establish direct TOR modulation as the sole mechanism underlying the longevity phenotype.

When we investigated whether the longevity-promoting effects of LSD required continuous exposure, we found that shorter treatment regimens (24, 48, 72, 96, and 120 hours) each produced a similar beneficial effect. Together, these results indicate that LSD can induce long-lasting physiological changes sufficient to promote lifespan extension even after limited exposure, a feature that may be especially promising in a therapeutic context.

A limitation of this study is the use of 5-fluorouracil to suppress progeny production. Although 5-FU was applied identically to all experimental groups, pharmacological suppression of progeny production can introduce confounding effects in lifespan studies, particularly in experiments involving dietary interventions. We therefore cannot exclude an interaction between 5-FU and either LSD treatment or the reduced-food condition. Confirming these findings in the absence of 5-FU, with progeny controlled manually, will be an important next step.

## Conclusion

In conclusion, our findings indicate that LSD extends lifespan and is associated with reduced age-associated physiological decline in *C. elegans*. This effect depends, at least in part, on serotonergic signaling and is accompanied by physiological and metabolic changes consistent with modulation of conserved nutrient-sensing pathways. Food-consumption assays did not detect a significant reduction under the conditions tested. Together, these results position LSD as a useful tool to investigate how serotonergic signaling interfaces with aging-related pathways in *C. elegans*.

## Supplementary Information

Below is the link to the electronic supplementary material.

Supplementary file1 (DOCX 223 KB)

## Data Availability

No datasets were generated or analysed during the current study.
