# Supplementary material for: Lysergic acid diethylamide extends lifespan in Caenorhabditis elegans
Source: Biogerontology. 2026 Oct 22;27(6):184. doi: 10.1007/s10522-026-10521-1 (PMC13690901; doi:10.1007/s10522-026-10521-1)
Supplement: Supplementary file 1 [file 10522_2026_10521_MOESM1_ESM.docx]

**Supplementary table 1 – Effects of LSD on lifespan**

| Strain | Treatment time | LSD concentration (nM) | Feeding condition | Number of animal  (LSD-/LSD+) | Number of experiments | Percentage change | *P* value |
| --- | --- | --- | --- | --- | --- | --- | --- |
| N2 (Wild Type) | Throughout adulthood | 100 | *Ad libitum* | 137/174 | 4 | 12.4 | 0.0321 |
| N2 (Wild Type) | Throughout adulthood | 200 | *Ad libitum* | 137/174 | 4 | 13.2 | 0.0055 |
| N2 (Wild Type) | Throughout adulthood | 250 | *Ad libitum* | 137/271 | 4 | 17.6 | 0.0026 |
| N2 (Wild Type) | 24h | 250 | *Ad libitum* | 215/240 | 4 | 5.6 | 0.0043 |
| N2 (Wild Type) | 48h | 250 | *Ad libitum* | 231/219 | 4 | 9.9 | 0.0007 |
| N2 (Wild Type) | 72h | 250 | *Ad libitum* | 202/199 | 4 | 11 | 0.0063 |
| N2 (Wild Type) | 96h | 250 | *Ad libitum* | 206/209 | 4 | 12.1 | 0.0058 |
| N2 (Wild Type) | 120h | 250 | *Ad libitum* | 210/238 | 4 | 11.1 | <0.0001 |
| N2 (Wild Type) | Throughout adulthood | 250 | *Caloric restriction* | 167/135 | 3 | -9.2 | 0.0072 |
| *ser-4* (ok512) | Throughout adulthood | 250 | *Ad libitum* | 254/212 | 4 | -7.7 | <0.0001 |
| *ser-1* (ok345) | Throughout adulthood | 250 | *Ad libitum* | 240/230 | 4 | -2.2 | 0.3580 |
| *ser-5* (vq1) | Throughout adulthood | 250 | *Ad libitum* | 130/180 | 3 | 0.5 | 0.9582 |
| *ser-7(tm1325)* | Throughout adulthood | 250 | *Ad libitum* | 122/216 | 3 | -3.3 | 0.0169 |
| *tph-1*(mg280) | Throughout adulthood | 250 | *Ad libitum* | 199/213 | 3 | 9.3 | 0.0002 |
| *raga-1* (ok386) | Throughout adulthood | 250 | *Ad libitum* | 224/230 | 4 | -11.6 | 0.0023 |
| *eat-2* (ad1116) | Throughout adulthood | 250 | *Ad libitum* | 131/154 | 4 | 3.5 | 0.2543 |

**Supplementary table 1. Summary of lifespan assays in *C. elegans*.** Lifespan assays were performed in *wild-type* (N2), *ser-1*(ok345), *ser-4*(ok512), *ser-5*(vq1), *ser-7*(tm1325), *tph-1*(mg280), *raga-1* (ok386) and *eat-2* (ad1116). Worms were exposed to 250 nM LSD either chronically (whole-life) or for defined durations (24–120 h). The number of animals analyzed per group (control or LSD-treated), number of biological replicates, and percentage change in lifespan relative to controls are shown. Survival analysis was performed using OASIS 2, and the percentage change corresponds to a late-survival time point defined as the time at which 25% of the animals remained alive. For the N2 dose-response experiment (100, 200, and 250 nM LSD), the three treatment groups were compared with the same shared control group (n=137). *P*-value was determined by comparing survival curves of treated and untreated animals of the same strain using the log-rank (Mantel–Cox) test.

**Supplementary table 2 – Effects of LSD on oviposition**

| Strain | Analysis | Number of animal  (LSD-/LSD+) | Number of experiments | *P* value |
| --- | --- | --- | --- | --- |
| N2 (Wild Type) | Time | 37/37 | 5 | <0.0001 |
|  | Treatment |  |  | 0.0083 |
|  | Time x Treatment |  |  | <0.0001 |

**Supplementary Table 2. Summary of oviposition assays in *C. elegans*.** Oviposition assays were performed in wild-type (N2) animals exposed to vehicle or 250 nM LSD. The number of animals analyzed per group (control/LSD-treated) and the number of independent biological experiments are indicated. Oviposition was assessed by quantifying the number of eggs laid over time. Data were analyzed using two-way ANOVA with time and treatment as factors. *P* values for the main effects of time and treatment, as well as the time × treatment interaction, are shown in the table.

**Supplementary table 3 – Effects of LSD on body size**

| Strain | Treatment time  (days) | Number of animal  (LSD-/LSD+) | Number of experiments | *P* value |
| --- | --- | --- | --- | --- |
| N2 (Wild Type) | 1 | 32/32 | 3 | 0.0056 |
| N2 (Wild Type) | 10 | 21/20 | 3 | 0.0380 |

**Supplementary Table 3. Summary of body size assays in *C. elegans*.** Body size assays were performed in wild-type (N2) animals exposed to 250 nM LSD for 1 or 10 days. The number of animals analyzed per group (control or LSD-treated), number of biological replicates, and *P* values are shown. Animal length was measured using LAS X software. Images were acquired using a Leica M165 FC stereomicroscope at 100× magnification *P* values were determined by comparing body size between treated and untreated using the Student's t-test.

**Supplementary table 4 – Effects of LSD on pharyngeal pumping**

| Strain | Comparison  (group 1 vs group 2) | Number of animal (group 1/ group 2) | Number of experiments | *P* value |
| --- | --- | --- | --- | --- |
| N2 (Wild Type) | Control D1 vs LSD D1 | 23/23 | 3 | 0.9969 |
| N2 (Wild Type) | Control D1 vs LSD D5 | 23/23 | 3 | 0.6923 |
| N2 (Wild Type) | Control D1 vs Control D5 | 23/24 | 3 | <0.0001 |
| N2 (Wild Type) | Control D5 vs LSD D5 | 24/23 | 3 | 0.0059 |
| N2 (Wild Type) | LSD D1 vs LSD D5 | 23/23 | 3 | 0.3392 |

**Supplementary Table 4. Summary of pharyngeal pumping assays in *C. elegans*.** Pharyngeal pumping assays were performed in wild-type (N2) animals exposed to vehicle or 250 nM LSD for 1 or 5 days. The number of animals analyzed per group (control/LSD-treated) and the number of independent biological experiments are indicated. Pharyngeal pumping frequency was determined by counting pharyngeal contractions for 10 seconds observation periods and converting the values to contractions per minute. *P* values correspond to the pairwise comparisons indicated in the table and were obtained using two-way ANOVA followed by Sidak’s multiple-comparisons test.

**Supplementary table 5 – Effects of LSD on food consumption**

| Strain | Treatment time  (days) | Number of animal  (LSD-/LSD+) | Number of experiments | *P* value |
| --- | --- | --- | --- | --- |
| N2 (Wild Type) | 1 | 80/80 | 4 | 0.6114 |
| N2 (Wild Type) | 5 | 80/80 | 4 | 0.1167 |

**Supplementary Table 5. Summary of food consumption assays in *C. elegans*.** Food consumption assays were performed in wild-type (N2) animals exposed to 250 nM LSD for 1 or 5 days. The number of animals analyzed per group (control or LSD-treated), number of biological replicates, and *P* values are shown. The remaining bacterial concentration was quantified by measuring optical density at 600 nm (OD_600_). *P* values were determined by comparing bacterial consumption between treated and untreated animals using Student’s *t*-test.

**Supplementary table 6 –Effects of LSD on lipofuscin accumulation and HLH-30 nuclear levels and PHA-4 expression**

| Strain | Treatment time  (days) | Number of animal  (LSD-/LSD+) | Number of experiments | *P* value |
| --- | --- | --- | --- | --- |
| N2 (Wild Type) Lipofuscin | 10 | 36/32 | 3 | 0.3248 |
| N2 (Wild Type)  Lipofuscin | 15 | 31/25 | 3 | <0.0001 |
| *pha-4::GFP* | 1 | 18/19 | 3 | 0.0055 |
| *pha-4::GFP* |  | 20/19 | 3 | 0.0378 |
| *pha-4::GFP* | 10 | 19/18 | 3 | 0.1115 |
| *hlh-30::GFP* | 1 | 28/24 | 3 | 0.9358 |
| *hlh-30::GFP* | 5 | 30/29 | 3 | 0.3939 |
| *hlh-30::GFP* | 10 | 28/26 | 3 | 0.0302 |

**Supplementary table 6. Summary of fluorescence-based assays.** Fluorescence-based assays were performed in animals exposed to 250 nM LSD or vehicle control. The number of animals analyzed per group, number of biological replicates, and *P* values are shown. Lipofuscin accumulation was quantified based on autofluorescence intensity, whereas PHA-4 levels were assessed by GFP fluorescence intensity in PHA-4::GFP reporter animals. HLH-30 nuclear localization was evaluated in HLH-30::GFP reporter animals by classifying animals according to the presence or absence of nuclear translocation. Statistical comparisons between treated and untreated animals were performed using Student’s t-test.

**Supplementary table 7 – Effects of LSD on lipid accumulation**

| Strain | Treatment time  (days) | Number of animal  (LSD-/LSD+) | Number of experiments | *P* value |
| --- | --- | --- | --- | --- |
| N2 (Wild Type) | 1 | 32/25 | 3 | <0.0001 |
| N2 (Wild Type) | 5 | 24/26 | 3 | 0.0278 |
| N2 (Wild Type) | 10 | 31/31 | 3 | 0.7055 |

**Supplementary table 7. Summary of lipid accumulation assays in *C. elegans*.** Lipid accumulation assays were performed in wild-type (N2) animals exposed to 250 nM LSD for 1, 5 or 10 days. The number of animals analyzed per group (control or LSD-treated), number of biological replicates, and *P* values are shown. Lipid accumulation was evaluated using Oil Red O staining. *P* values were determined by comparing lipid accumulation between treated and untreated using the Student's t-test.

**Supplementary table 8 – Effects of LSD on vulval development**

| Strain | Treatment time  (days) | Number of animal  (LSD-/LSD+) | Number of experiments | *P* value |
| --- | --- | --- | --- | --- |
| N2 (Wild Type) | 2 | 53/56 | 4 | 0.0022 |

**Supplementary Table 8. Summary of vulval development assays in *C. elegans*.** Vulval development assays were performed in wild-type (N2) animals exposed to 250 nM LSD for 48 hours. The number of animals analyzed per group (control or LSD-treated), number of biological replicates, and *P* values are shown. Vulval development was evaluated after LSD exposure. *P* values were determined by comparing LSD-treated and untreated animals of the same strain using the Student's t-test.

**Supplementary figure 1.**

**Supplementary figure 1**. **Lifespan response to different LSD concentrations**. Percentage survival of treated with 100 or 200 nM LSD (orange and red, respectively) or untreated animals (black) over time. Data presented as Kaplan-Meier survival curves, and group differences analyzed using the Log-rank test. n=4, with at least 130 animals per condition.
